# Risk Factors and Surgical Outcomes of Diabetic Foot in Diabetic Patients at King Fahad University Hospital

**DOI:** 10.7759/cureus.32457

**Published:** 2022-12-12

**Authors:** Yasser A Elghoneimy, Ali A Alkabah, Hasheem M Alalsayedsalih, Ali J Almanyan, Hassan A Alibrahim, Mostafa H Albokamsin, Shadi A Alshammary, Fahd A Makhdom

**Affiliations:** 1 Department of Cardiac Surgery, King Fahad University Hospital, Dammam, SAU; 2 College of Medicine, Imam Abdulrahman Bin Faisal University, Dammam, SAU; 3 Department of General Surgery, King Fahad University Hospital, Dammam, SAU

**Keywords:** diabetic foot complications, diabetic gangrene, prevalence of diabetic foot ulcer, diabetes mellitus, diabetic foot

## Abstract

Background: Diabetes mellitus affects a large number of the population worldwide. One of the most important complications is diabetic foot ulcers, which are debilitating to the patient and the health care system.

Objectives: To assess the risk factors leading to diabetic foot complications and the surgical outcome of management of patients affected by diabetic foot at King Fahd University Hospital.

Methods: This is a retrospective case series study conducted at King Fahd University Hospital. The study included 52 patients who were admitted from the period 2007 to 2017 with cases of diabetic foot ulcers. The study includes a review of medical charts for diabetic foot patients including demographics, diabetic profile, associated comorbidities, clinical signs, laboratory investigations and surgical outcome.

Results: Our study shows that diabetic foot patients had comorbidities like hypertension at 69.2% and hyperlipidemia at 55.8%. Regarding the association between comorbidities and surgical outcomes, we did not find any significance. Furthermore, we found that patients with gangrene were more likely to undergo amputation. Patients who underwent amputation had hemoglobin A1c (HbA1c) of 10.3 ± 2.4% and random blood sugar (RBS) of 259 ±107 mg/dl, indicating poor diabetic control.

Conclusion: Diabetic foot is the result of poor blood sugar control. Surgical intervention plays an important role in the management of diabetic foot. Depending on the presentation, patients may undergo surgical debridement, revascularization, or amputation. We found that patients who developed gangrene were more likely to undergo amputation.

## Introduction

Diabetic foot disease can be defined as infection, ulceration, or destruction of tissues of the foot of a person who is diagnosed with diabetes mellitus (DM) and which is usually associated with neuropathy and/or peripheral arterial disease in the lower extremity [1]. A usual presentation involves a trauma to the foot due to loss of sensation, along with poor healing and neglect. This may further develop into ulcers and gangrene depending on how early the presentation was. When medical management for diabetic foot is insufficient, advanced surgical management like amputation will be required [2]. Diabetic foot patients have higher mortality rate than patients without diabetic foot [3]. Moreover, a large number of diabetic foot patients will need surgical intervention, which will increase the rate and the duration of inpatient hospitalizations. The amputation rate is rising, which serves as a major concern that leads to the importance of implementing all precautions and safeguarding measures for foot care [4]. Therefore, this study aims to estimate the risk factors and the surgical outcomes of diabetic foot. Estimating the risk factors and surgical outcomes of diabetic foot can be assessed in the management process. 

## Materials and methods

This is a retrospective observational study done at King Fahad University Hospital in Al Khobar, Saudi Arabia. The study included patients admitted from 2007 to 2017 as cases of diabetic foot which includes ulcer, gangrene, or vascular complications. Data collection was done through the hospital’s information system (Quadramed) which contained the patients’ medical files. All patients who did not meet the criteria were excluded from the study. Exclusion criteria include patients with incomplete records or patients who were admitted for other medical complaints, pediatric age group and traumatic patients. The total number of diabetic foot patients admitted from the period 2007 to 2017 was 198 patients. The number of patients who met the inclusion criteria was 52 patients. 

All-important data that can contribute to the risk of diabetic foot development were collected and analyzed including patient's identification data, associated comorbidities, details of the diabetic course of illness like type of diabetes, presence of previous ulcer, micro and macrovascular complications with their prevalence, and surgical outcome including use of debridement, revascularization, and amputations.

Data collection and analysis of laboratory reports were done for interpretation of diabetic course and severity including fasting blood sugar (FBS), hemoglobin A1c (HbA1c), random blood sugar (RBS), blood urea nitrogen (BUN), and lipid levels. Physical examinations for neuropathy, retinopathy, and peripheral vascular disease were collected. In addition, more local and specific exams were done for common conditions associated with diabetes like cellulitis callus, Charcot foot, and the presence of gangrene and ulcers. The outcome was determined based on thorough assessment including labs, imaging, and clinical assessment of limb viability to determine whether to save the limb by doing a revascularization therapy or amputation. Moreover, we included in our study a cross relation to see the association among the means of the lab results including HbA1c, RBS, BUN, total cholesterol (TC) and the surgical outcomes whether it is amputation, revascularization, or debridement. Those associations were analyzed to have a more detailed view on what affects the outcome of diabetic foot. 

Data analysis was done by the IBM SPSS version 21 (IBM Corp., Armonk, NY, USA). Categorical values were described as frequency and percentage. Numerical variables were used to calculate the mean and the standard deviation. Chi square test was used for comparison of the observed and expected results. To interpret the significance of the results a p-value of less than 0.05 was considered significant. 

## Results

Table 1 shows the descriptive demographic information of the study sample. A total number of 52 patients were covered with a mean age of 64 ± 12 years. The mean weight was 83.27 ± 17.9 kg, and the mean height was 1.62 ± 0.07 m.

**Table 1:** Demographics (A) (descriptive)

Demographics	Minimum	Maximum	Mean
Age	33	94	64.038
Weight	55.0	123.0	83.270
Height	1.520	1.77	1.6269

Table 2 shows the demographic information of the study sample. Eighteen patients were females which represents 34.6%, and 34 were males which represents 65.4% of the total participant’s number. 

**Table 2:** Demographics (B), comorbidities, diabetes complications, and local examination HTN: hypertension

Demographics (B)		Total	Females	Males
	Gender	52	18 (34.6%)	34 (65.4%)
	Saudi	41 (78.8%)	16 (88.9%)	25 (73.5%)
	Non-Saudi	11 (21.2%)	2 (11.1%)	9 (26.5%)
comorbidities	HTN	36 (69.2%)	17 (94.4%)	19 (55.9%)
	Hyperlipidemia	29 (55.8%)	13 (72.2%)	16 (47.1%)
	Chronic kidney disease	14 (26.9%)	7 (38.9%)	7 (20.6%)
	Coronary artery disease	9 (17.3%)	4 (22.2%)	5 (14.7%)
	Thyroid Disease	1 (1.9%)	1 (5.6%)	0 (0%)
diabetes complications	Neuropathy	31 (59.6%)	10 (55.6%)	21 (61.8%)
	Nephropathy	22 (42.3%)	9 (50.0%)	13 (38.2%)
	Retinopathy	18 (34.6%)	8 (44.4%)	10 (29.4%)
	Coronary heart disease	9 (17.3%)	4 (22.2%)	5 (14.7%)
local examination	Callus	2 (3.8%)	1 (5.6%)	1 (2.9%)
	Charcot foot	0 (0%)	0 (0%)	0 (0%)
	Peripheral neuropathy	29 (55.8%)	9 (50.0%)	20 (58.8%)
	Ulcer	43 (82.7%)	13 (72.2%)	30 (88.2%)
	Gangrene	21 (40.4%)	6 (33.3%)	15 (44.1%)
	Cellulitis	13 (25%)	6 (33.3%)	7 (20.6%)

Comorbidities found in the diabatic foot patients were as follows: hypertension (69.2%) and hyperlipidemia (55.8%) were found in more than half of the patients, followed by chronic kidney diseases (26.9%), coronary artery diseases (17.3%) and thyroid disease (1.9%).

Diabetes complications included neuropathy (31; 59.6%), followed by nephropathy (22; 42.3%) and retinopathy (18; 34.6%). Nine (17.3%) of the patients were noted to have coronary heart disease. As evidenced by the results (Table 2) on mean, patients presented with more than one diabetic complication alongside diabetic foot.

Upon local examination, most patients presented with foot ulcer (43; 82.7%), 29 (55.8%) presented with peripheral neuropathy, 21 (40.4%) with gangrene, 13 (25%) with cellulitis and only two (3.8%) presented with callus. As evidenced by the result (Table 2) patients on average were presented with more than one finding.

Table 3 shows history information for 52 diabetic foot patients including their type of DM, duration of DM, and previous ulcer. In this research most diabetic foot patients had type 2 DM (96.2%). In reference to the duration of illness in patients with type 2 DM and the development of ulcers, about 1.9% had the problem of the diabetic foot during the first 10 years of illness. During the interval of 10-20 years 21.2% of the patients had the disease, 13.4% of patients had the disease for more than 20 years. Patients with type 1 DM were included in the missing data for the duration of illness. In addition, more than half of the patients did not have previous ulcers (61.5%).

**Table 3:** Diabetic profile DM: diabetes mellitus

Diabetic profile		Total	Females	Males
Type of DM	DM1	2 (3.8%)	0 (0%)	2 (5.9%)
	DM2	50 (96.2%)	18 (100%)	32 (94.1%)
Duration of DM (in years)	<10	1 (1.9%)	0 (0%)	1 (6.1%)
	10-20	11 (21.2%)	2 (66.6%)	9 (56.5%)
	>20	7 (13.4%)	1 (33.4%)	6 (37.4%)
	Missing	35 (63.5%)		
Previous ulcer	YES	20 (38.5%)	5 (27.8%)	15 (44.1%)

Table 4 shows the surgical outcome for 52 diabetic foot patients including the process of amputation, revascularization, and debridement. Note that some patients may need more than one procedure to recover, so the frequency is not the same as the number of patients. 

**Table 4:** Outcomes HTN: hypertension

	Within	Amputation		Revascularization		Debridement	
		N (%)	P	N (%)	P	N (%)	p
Gender	Females	9 (50%)		0 (0%)		11 (61.1%)	
	Males	17 (50%)		4 (11.8%)		16 (47.1%)	
	Total	26 (50%)	1.00	4 (7.7%)	0.13	27 (51.9%)	0.33
Comorbidity	HTN	19 (73.1%)	0.54	2 (50%)	0.38	16 (59.3%)	0.10
	Hyperlipidemia	16 (61.5%)	0.40	1 (25%)	0.19	14 (51.9%)	0.55
	Chronic kidney disease	9 (34.6%)	0.21	0 (0%)	0.20	7 (25.9%)	0.86
	Thyroid Disease	0 (0%)	0.31	0 (0%)	0.77	0 (0%)	0.29
Diabetic complications	Neuropathy	18 (69.2%)	0.15	2 (50%)	0.68	14 (51.9%)	0.23
	Nephropathy	13 (50%)	0.26	1 (25%)	0.46	10 (37%)	0.42
	Retinopathy	10 (38.5%)	0.56	0 (0%)	0.13	6 (22.2%)	0.05
	Coronary heart disease	6 (23.1%)	0.27	0 (0%)	0.34	2 (7.4%)	0.05
	Cerebrovascular disease	0 (0%)		0 (0%)		0 (0%)	
	Previous ulcer	14 (53.8%)	0.02	1 (25%)	0.56	11 (40.7%)	0.72
Local examination	Callus	0 (0%)	0.14	0 (0%)	0.67	0 (0%)	0.13
	Charcot foot	0 (0%)		0 (0%)		27 (100%)	
	Peripheral neuropathy	17 (65.4%)	0.16	3 (75%)	0.42	15 (55.6%)	0 .97
	Ulcer	22 (84.6%)	0.71	4 (100%)		23 (85.2%)	0.62
	Gangrene	17 (65.4%)	0.00	2 (50%)	0.68	10 (37%)	0.60
	Cellulitis	9 (34.6%)	0.10	0 (0%)	0.22	6 (22.2%)	0.63

As illustrated in table 4, there were 26 (50%) patients who had to go through amputation, four (7.7%) had revascularization and 27 (51.9%) underwent debridement. There was no significance when it came to the relation between gender and outcome.

As for comorbidities, the number of hypertensive patients who underwent amputation was 19 (73.1%), two (50%) revascularization and 16 (59.3%) debridement. Following hypertension, hyperlipidemia accounted for 16 (61.5%) of the patients who underwent amputation, one (25%) for revascularization and 14 (51.9%) for debridement. After that, chronic kidney disease was found in nine (34.6%) of amputated patients, and seven (25.9%) of debrided patients. There was no evident significance in the relation between comorbidities and outcome.

When it came to diabetic complications, the most common complication was neuropathy, around 18 (69.2%) patients who underwent amputation had neuropathy, two (50%) revascularization and 14 (51.9%) debridement. Followed by nephropathy, 13 (50%) of patients who underwent amputation had nephropathy, one (25%) revascularization and 10 (37%) debridement. Ten (38.5%) patients who underwent amputation had retinopathy, 0 (0%) revascularization and six (22.2%) debridement. For other complications like coronary heart disease, six (23.1%) patients who underwent amputation had coronary heart disease, 0 (0%) revascularization and two (7.4%) debridement. Also, multiple patients had previous ulcers. Fourteen (53.8%) of patients who underwent amputation had previous ulcers, one (25%) revascularization and 11 (40.7%) debridement. 

In the relation between debridement and retinopathy, a statistically significant p value was noted (0.05). Also, in the relation between debridement and coronary heart disease the same value was noted (0.05). Another statistically significant p value was shown in the relation between amputation and the presence of previous ulcers (0.02). 

On local examination, a high number of patients had ulcers, 22 (84.6%) of patients who underwent amputation had ulcer on local exam, four (100%) revascularization and 23 (85.2%) debridement. Also, peripheral neuropathy was observed in multiple patients, 17 (65.4%) patients who underwent amputation had peripheral neuropathy on local exam, three (75%) revascularization and 15 (55.6%) debridement. Around 17 (65.4%) of patients who underwent amputation had gangrene on local exam, two (50%) revascularization and 10 (37%) debridement. Also, nine (34.6%) of patients who underwent amputation had cellulitis on local exam, 0 (0%) revascularization and six (22.2%) debridement. In the relation between amputation and gangrene a statistically significant p value was noted (0.00).

Table 5 shows the mean lab values which include HbA1c, RBS, BUN, and TC or the diabetic foot patients who underwent surgical management, divided into three groups (amputation, revascularization, and debridement). Regarding the HbA1c, all categories show notable high readings ranging from 10.3% ± 2.4 in amputation to 10.7% ± 1.2 in revascularization which is much higher than the desired value in these patients. For RBS, two groups showed high mean values, the amputation group 259mg/dL ± 107 and the debridement group 291mg/dL ± 123, the mean value for the revascularization group was 122mg/dL ± 37. All these values indicate that the majority of the diabetic foot patients had poor blood sugar control

**Table 5:** Mean laboratory values associated with outcomes FBS: fasting blood sugar, HbA1c: hemoglobin A1c, RBS: random blood sugar, BUN: blood urea nitrogen, TC: total cholesterol

Laboratory (mean)	Amputation	Revascularization	Debridement
HBA1C %	10.313	10.700	10.352
RBS mg/dL	259.91	122.33	291.35
BUN mg/dL	24.38	23.0	22.33
TC mg/dL	161.38	120.0	161.95

## Discussion

The goal of this study was to evaluate the outcomes of diabetic foot in diabetic patients, categorizing them as amputation, revascularization, and debridement treatments. The study was also planned to investigate the influence of several risk variables on diabetic foot outcomes, including patients’ gender, comorbidities, diabetic complications such as peripheral neuropathy, and prior ulcers.

Identifying variables that are linked to the development of diabetic foot is critical in clinical practice, to prevent its occurrence among high-risk diabetic patients. Hypertension is one of the powerful predictors and modifiable risk factors for the macrovascular and microvascular complications of diabetes. Previous research found that diabetic foot was linked to hypertension [5], which is consistent with our findings. Furthermore, hypertension prevalence is twice in diabetic individuals compared to non-diabetics [6]. In addition, in our results, more than half of diabetic patients with hypertension had an amputation, two patients had revascularization, and 16 patients had debridement. Arterial hypertension influences foot ulcer and gangrene development by increasing arterial wall stiffness and plaque formation in the lower extremities, which are the main reasons for the co-existence of hypertension and the aforementioned outcomes [5]. Other comorbidities like hyperlipidemia were found to be common among the group, at 29 patients. Moreover, 14 diabetic patients suffered from chronic kidney disease, and all of these comorbidities factors are related to a worse outcome [7]. 

A history of previous ulcers raises the chance of additional lesions and carries a risk of lower extremity amputation, which is equivalent to those who have had their first ulcer [8,9]. According to our study, recurrence of ulcers was found in 38% of patients, 14 patients of which had to be amputated, one had to be revascularized, and 11 had to be debrided. This discovery is critical because foot ulcers may be avoided with proper education on foot care and attention to footwear [10,11]. A strong link exists between amputation and ulcer recurrence according to our findings.

One of the key risk factors for all diabetic foot issues is peripheral neuropathy. All participants who did not have peripheral neuropathy had healed ulcers without sequelae according to our results. This substantial link between peripheral neuropathy and diabetic foot issues was also found in a study where 33% of diabetic patients had chronic nerve compression [12]. 

In our study, 17 patients had to be amputated, three had to be revascularized, and 15 had to be debrided. Improving glycemic control, and patient education about foot care are critical steps in reducing the burden of diabetic foot and its impact on the quality of life.

Analysis and interpretation of lab results are an essential part of assessment of disease progression, severity and for establishing a diagnosis, especially for diabetic foot. HbA1c was high which necessitated re-evaluation of the medical management, drug regimen and the need for inclusion of lifestyle modifications. Moreover, the RBS level was also high, and if not controlled it can lead to hyperglycemic hyperosmolar state and severe dehydration. 

According to our findings, patients who did amputation, revascularization and debridement had a mean HbA1c of 10.3%, 10.7%, and 10.3% respectively. All HbA1c levels are very high above the 10% mark, which may indicate the relation between high HbA1C and surgical intervention in any form. In addition, we measured BUN levels to monitor the progression of kidney disease and to avoid any drug-related nephrotoxicity. Elevated BUN showed an involvement in the outcome; the highest recorded value was 24.3mg/dL which was associated with amputation. Also, it is crucial to monitor cholesterol levels specifically in diabetic patients, due to the increased risk of developing macrovascular complications that affect the heart and the peripheries and try to keep it at normal values [13]. Patients with higher total cholesterol levels are more vulnerable to macrovascular complications in the form of ulcers and gangrene [14]. 

Diabetic complications can show the progression of the disease and can be a factor in prognosis [15]. Nephropathy was common with 22 of the patients being affected. Multiple studies showed that patients with end-stage renal disease are at higher risk of complications like amputation, gangrene, and ulceration [16-18]. A study involving 430 patients found that the presence of retinopathy placed the patients at an increased risk of developing diabetic foot ulcers. We found around one-third of our patients had retinopathy as a diabetic complication, so those patients are at risk [19]. 

Other findings that can increase the risk of amputation are the presence of ulcers, which affected a very high number of the group (43), and gangrene which was present in 21 patients in examination [20]. A significant relation was established between both patients who had previous ulcers and patients who had gangrene upon examination with the need for amputation later on as an outcome, which was also shown in other studies. This would lead us to believe that prevention of both the development of gangrene and ulcers is of great importance [21].

In our view, we advise that primary health care practitioners promote their participation in diabetic foot screening by employing foot screening instruments, as well as patient education. The importance of early identification and care of diabetic foot cannot be ignored, because diabetic patients are at risk of diabetic foot complications, such as the elderly and those with poorly managed or long-term DM, bigger ulcers, and peripheral neuropathy, should be given extra attention. The patient's age, diabetes duration, glycemic control, ulcer size, and comorbidities such as peripheral neuropathy can all be used to predict the patient's diabetic foot result. Larger research is needed in the future to further assess diabetic foot consequences in Saudi Arabia.

Limitations and recommendations

The study is a retrospective with a small sample size, due to limited information about the duration of the disease and the oral hypoglycemic agents due to insufficient data in the system. There are many co-founding factors that might affect the surgical outcome; thus, we suggest a propensity-matched study to be performed prospectively to get more accurate results with a larger sample size.

## Conclusions

in conclusion, the study showed that diabetic foot patients had comorbidities like hypertension (69.2%) and hyperlipidemia (55.8%). However, there is no significant relationship between comorbidities and surgical outcomes. Furthermore, laboratory investigations like HbA1c and RBS were also high in diabetic foot patients, which correlate with poor blood sugar control. Finally, as per the results discovered in the study, one of the causes of diabetic foot is poor blood sugar control. The study showed that surgical interventions like amputation, revascularization or debridement might help to manage or recover some diabetic foot patients. However, cases that developed gangrene were more likely to undergo amputation as there is a vital relationship between them.
